# Characteristics of polyclonal anti-interferon-gamma autoantibodies and novel diagnostic strategies: A prospective cohort study of new biomarkers

**DOI:** 10.1016/j.jtauto.2025.100292

**Published:** 2025-05-15

**Authors:** Xidong Wang, Feng Ye, Hongling Liu, Shaoqiang Li, Jinglu Yang, Xue Yu, Yilei Hui, Yongming Li, Yangqing Zhan, Yan Wang, Jing Liu, Zhengtu Li

**Affiliations:** a State Key Laboratory of Respiratory Disease, National Clinical Research Center for Respiratory Disease, Guangzhou Institute of Respiratory Health, The First Affiliated Hospital of Guangzhou Medical University, Guangzhou, 510120, China; b Department of Infectious Diseases, Third Affiliated Hospital of Sun Yat-Sen University, Guangzhou, China

**Keywords:** *Anti*-γ interferon autoantibody, Chromatographic method, ELISA method, IgG, Isolation and purification

## Abstract

**Background:**

*Anti*-γ interferon autoantibody (AIGA) syndrome is a widespread and grossly underestimated immunodeficiency disorder characterized by high mortality rates and a lack of standardized diagnostic methods. A highly accurate AIGA biomarker that meets the requirements of absolute quantification is urgently needed to enable the early diagnosis and treatment monitoring of the disease. In our study, we aimed to identify the primary components of AIGAs, determine their function, and develop a novel diagnostic method.

**Methods:**

Immune repertoire sequencing and a retrospective antibody subtype index analysis were performed for typical patients. Affinity chromatography was used to isolate and purify IgGs from AIGAs in the plasma of AIGA(+) patients. The clinical application value of chromatography for testing AIGAs was evaluated in a prospective clinical cohort.

**Results:**

A total of 114 eligible subjects were enrolled. Immune repertoire sequencing revealed that 74 % of the AIGA(+) patients had IgG clone types, with the somatic hypermutation (SHM) analysis being the most informative. We isolated AIGAs from the blood and interpreted their affinity and major components completely. Based on the results of this prospective cohort study, AIGAs, an absolute quantitative biomarker, were significantly better than the ELISA method (Delong test, P = 0.0018).

**Conclusions:**

Patients with AIGA syndrome have abnormally elevated IgG levels, with IgG3 subtypes dominating. The disorder is characterized by the rapid acquisition of polyclonal AIGAs. The obtained AIGAs had a good neutralization capacity and potential as absolute quantitative biomarkers.

## 1 Introduction

In recent years, anticytokine autoantibodies have attracted the attention of researchers as important etiologic agents of infection caused by many life-threatening pathogens [[1], [2], [3], [4]]. *Anti*-γ interferon autoantibody (AIGA) syndrome is a newly discovered type of anticytokine autoantibody disease. Disruption of the function of interferon-gamma (IFN-γ) prevents the production of inducible antimicrobial proteins, thereby inhibiting the ability of the human immune system to clear pathogens [5,6]. Studies have shown that the interferon signaling pathway may be downstream of the disease, leading to the occurrence of infections [[7], [8], [9]]. Disorders in the interferon signaling pathway may directly induce autoimmune diseases. For example, through lipid rafts, these disruptions can affect interferon pathway-mediated regulation of antigen presentation, cytokine secretion, and activation of T cells and B cells, thereby exerting significant effects on immune cell function [[10], [11], [12], [13]].

The presence of AIGAs has been shown to be associated with opportunistic infections caused by a variety of intracellular pathogens, including *Talaromyces marneffei* (TM) and nontuberculous mycobacteria (NTM), with a pathogen spectrum that includes 41 pathogens. Upon disease development, AIGAs are difficult to eliminate, are prone to causing recurrent and even life-threatening episodes, and are associated with a high incidence of double and multiple infections, accounting for 46.7 % of cases [14,15]. The lack of diagnostic specificity and the difficulty of treatment impose a heavy medical burden on patients with AIGAs and society in China. In clinical practice, highly accurate AIGA biomarkers that meet the requirements of absolute quantification are urgently needed to achieve early diagnosis and treatment monitoring of diseases.

A clear understanding of the composition of AIGAs is lacking. Lin et al. found that IgG was the major component of antibodies, which was confirmed through the successful isolation of monoclonal AIGAs from NTM patients [5]. However, a consensus has not been reached on the status of IgG isoforms in AIGAs. The IgG4 and IgG1 isoforms are the most commonly reported, and some IgG3 isoforms have also been reported. Structural explorations are just beginning. A study in Taiwan revealed that the artificial elimination of a single binding site on IFN-γ in the presence of AIGAs did not fully restore the function of its downstream components. The multiepitope binding of antibodies poses difficulties for molecular diagnostics, in which antigen‒antibody conjugation is the main technique. ELISAs for AIGAs have been performed with different commercially available ELISA antibodies for AIGAs, with large differences in titers between patients. ELISA has been used as a screening test, and a positive ELISA result does not indicate the presence of neutralizing activity [[16], [17], [18]]. Affinity chromatography is expected to address the problem of multiple binding sites for AIGAs, and some researchers have performed IFN-γ affinity purification of plasma from healthy individuals to isolate substances that inhibit downstream IFN-γ signaling in vitro. However, the product could not be enriched and further purified due to technical limitations, and its specific composition was not determined.

Based on this technical foundation and the needs of clinical practice, we propose a combination of high-throughput sequencing and basic experiments. Immune repertoire sequencing revealed elevated levels of IgG subtypes in patients. IFN-γ autoantibodies were purified via tandem affinity chromatography, functionally characterized, and subtyped. A prospective clinical cohort was established to validate the sensitivity and specificity of the AIGA biomarkers and to determine the feasibility of this method in clinical practice.

## 2 Methods

### 2.1 Patient enrollment and specimen collection

#### 2.1.1 Prospective clinical cohort

The research subjects enrolled as the prospective cohort of patients with AIGA syndrome were from a multicenter, prospective clinical study in China (ChiCTR2000029306). The patients included were those who were diagnosed with TM infection and were divided based on the presence of AIGA syndrome. The time range was from January 1, 2020, to December 31, 2023. All patients provided informed consent. Clinical samples, including serum and blood treated with EDTA, were obtained in accordance with routine clinical procedures. The samples were stored between 2 °C and 8 °C for no more than 48 h until they were ready for shipment to the central laboratory for processing.

#### 2.1.2 *Anti*-IFN-γ autoantibody (AIGA) syndrome [19,20]

The presence of AIGA syndrome-causative agents such as TM or NTM is commonly associated with pathogen coinfection, and the diagnosis is confirmed by the presence of a prolonged disease, dissemination of the infection, and a positive neutralization capacity in the AIGA neutralization assay (Bio-Layer Interferometry method and flow-through method).

##### 2.1.2.1 Control group

Patients with AIGA-negative *Talaromyces marneffei* infection are referred to as the TAIGA(−) group, and patients with AIGA-negative nontuberculosis mycobacteria infection are referred to as the NAIGA(−) group; all of these patients are comprehensively referred to as the AIGA(−) group. Healthy controls were included from the same community as the HC group.

## 3 10x Genomics immune repertoire and data analysis

The samples were processed using the Chromium single-cell V(D)J reagent kit (10x Genomics, PN-1000165)according to the protocol provided by 10x Genomics. We simultaneously profiled the immune repertoire (B-cell receptor, BCR) and gene expression from the same cells to enable the correlation of the clonotype with the corresponding cellular subtype.

## 4 Isolation and purification of AIGAs with chromatography

### 4.1 Isolation and purification of polyclonal autoantibodies

EDTA anticoagulant tubes containing patient blood samples were centrifuged at 800×*g* for 5 min to extract the upper plasma layer, and the supernatant was removed after the centrifugation of the plasma at 12000×*g* for 5 min. Albumin was removed with an Albumin Serum Removal Kit (Thermo Scientific™, 85160) using binding buffer (ECOTOP | ED-8706-500 ml), elution buffer (ECOTOP | ED-8707-500 ml) and a HiTrap Protein G HP (cytiva, 1 ml) column for the enrichment of IgG components and pH correction. Finally, the polyclonal AIGA solution was obtained via separation and purification using an assembled AIGA affinity chromatography column. The AIGA affinity chromatography column was assembled using a HiTrap NHS-activated column (Cytiva, 1 ml) coupled with 1 mg of IFN-γ (Sino Biological, 11725-HIS). Binding was performed using acidification solution (ECOTOP | ED-8701-500 ml), followed by three cycles of rinsing with blocking buffer (ECOTOP | ED-8702-500 ml) and wash buffer (ECOTOP | ED-8703-500 ml), with the pH adjusted to neutral for subsequent use. The IgG eluent was then adjusted to neutral pH using binding buffer (ECOTOP | ED-8706-500 ml) and elution buffer (ECOTOP | ED-8707-500 ml). Finally, the solution was neutralized to pH 7.0 using a 10 kDa (Merck Amicon® Ultra | UFC9010) ultrafiltration tube and PBS. Biolayer interferometry was used to perform affinity and competitive binding experiments.

### 4.2 Identification of AIGAs

A gradient dilution of the bovine serum albumin (BSA) standard was used to prepare BCA working solutions. The protein standards and the protein samples to be tested were added to a microtiter plate or test tube at each dilution, and the BCA working solution (the proportion of which was determined according to the instruction manual of the kit) was added and mixed. After the samples were sealed, they were incubated at 37 °C for 30–60 min and then cooled to room temperature, and the absorbance value at 562 nm was measured, with a blank used as a control. The absorbance at 562 nm of each standard and protein sample was measured by subtracting the average absorbance at 562 nm of the blank standard from the absorbance at 562 nm of the standard or sample.

## 5 Functional verification of AIGAs

### 5.1 The binding force of AIGAs

#### 5.1.1 Competitive binding assays based on biological layer interferometry (BLI)

The competitive binding of antibodies was determined on a ForteBio Octet Red96 system (Pall Forte BioCorporation, Menlo Park, CA) via a tandem-format sorting assay. Biotinylated proteins were loaded into the sensor (ForteBio, Cat. 18–5019). The sensor was then exposed to 30 μg/mL primary antibody or PBST for 300 s and then to 30 μg/mL secondary antibody for 300 s.

#### 5.1.2 Biolayer interferometry (BLI)-based affinity test

A chip was mounted according to the standard operating procedures of the OpenSPR™ instrument, activated, the functionalization of the chip surface was completed, and the ligand was prepared. Ligands for immobilization were diluted with activation buffer and injected, and the baseline was observed for 5 min to ensure stability. A high concentration of analyte was injected to confirm ligand activity and to confirm the approximate maximum binding capacity of the surface. The flow rate was increased to 150 μL/min, and the appropriate regeneration buffer was injected to remove the analyte.

## 6 Statistical analysis

Statistical analyses were performed using GraphPad Prism 6 (GraphPad Software Inc., La Jolla, CA, USA). Continuous variables are presented as the medians with interquartile ranges (IQRs). One-way ANOVA (Kruskal–Wallis test with Dunn's post hoc test) was used to compare data among different groups of participants. Normality was assessed using the Shapiro‒Wilk test (p > 0.05), and homogeneity of variance was confirmed via Levene's test. Parametric tests (Student's *t*-test) were applied for normally distributed data; otherwise, nonparametric alternatives (Mann‒Whitney *U* test) were used. Categorical variables were compared using Fisher's exact tests, as appropriate. A p value < 0.05 was considered to indicate statistical significance.

## 7 Results

1 Clinical features, diagnosis, and experimental flow chart for the prospective cohort

**Fig. 1:**
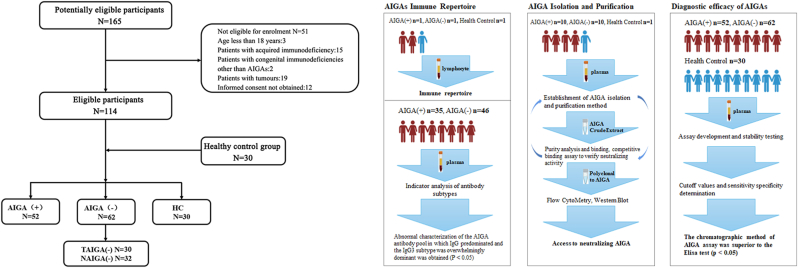
Patient enrollment process and experimental design. A: Patient enrollment flowchart. A total of 165 patients were screened for the study, and 51 patients were excluded, resulting in a total of 114 eligible subjects enrolled in this study, including 52 patients with AIGAs (AIGA(+) group), 30 patients with AIGA-negative *Talaromyces marneffei* infection (TAIGA(−) group) and 32 patients with AIGA-negative nontuberculosis mycobacteria infection (NAIGA(−) group), along with another 30 patients in the HC group. B: Distribution of clinical samples tested. Different numbers of patients were involved in different steps, and the study was divided into determining the AIGA immune repertoire and isolating AIGAs and determining their functions.

From January 1, 2020, to December 31, 2023, 165 subjects were screened for the study, and 51 patients who were not eligible for enrollment were excluded, resulting in a total of 114 eligible subjects enrolled in this study, including 52 patients with AIGAs (AIGA(+) group), 30 patients with AIGA-negative *Talaromyces marneffei* infection (TAIGA(−) group) and 32 patients with AIGA-negative *nontuberculosis mycobacteria* infection (NAIGA(−)), totaling 62 patients (AIGA(−) group). Another 30 healthy controls were included from the same community (HC group) (Fig. 1A).

2 VDJ immunohistochemistry suggested abnormally elevated IgG levels and a reduced immune repertoire diversity in AIGA(+) patients

**Fig. 2:**
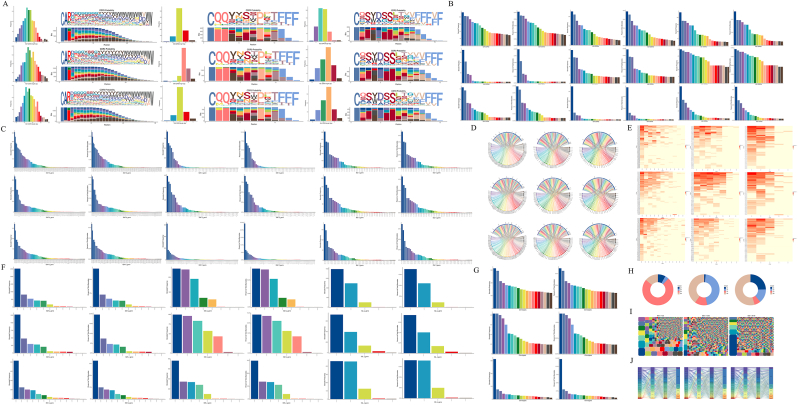
Demonstration of the VDJ immune repertoire (with the BCR). The groups from top to bottom are the TMP, TMN, and HC groups. A: Distribution of the amino acid sequence length, logo plot, and amino acid frequency stack plot for each group. B: Clonal distribution frequency of IGH, IGK, and IGL for each group. C: Distribution of the frequency of V genes in each group. D, E: Circle and heatmap representation of the V‒J paired combination frequency for IGH, IGK, and IGL in each group. F: Distribution of the cell frequency for J genes for IGH, IGK, and IGL in each group. G: Frequency distribution of clonotypes for each group. H: Isotype statistics for BCR. I: Clonotype dendrogram and diversity index (D50) reflecting the state and diversity of the immune process. J: Sankey diagram showing the distribution of clonotype V‒J gene chains.

We focused on the AIGA(+) patients’ BCR immune repertoire. The distribution of the CDR3 amino acid sequence (IGH, IGK, and IGL) lengths in the TMP group deviated significantly from normal (Fig. 2A). However, the proportion of amino acids in sequences of different lengths was similar across groups. The statistical analysis of the abundance of the CDR region in IGH revealed that the TMP group presented a significant number of mutations with a copy number of 100 or more, with the frequency of individual mutations fluctuating between 0.5 % and 2.5 % in the repertoire. Additionally, the TMN group presented a single high-mutation type at a proportion of 2.5 % (Fig. 2B and C). Finally, an analysis of the diversity index revealed that the D50 values for the HC, TMN, and TMP groups were 24.4, 15.03, and 1.52, respectively (Fig. 2I). The diversity of the TMN group differed significantly, with the clone types consisting mainly of several multiclone types and some small clone types, with some clone types having a distribution proportion exceeding 3 %.

**Fig. 3:**
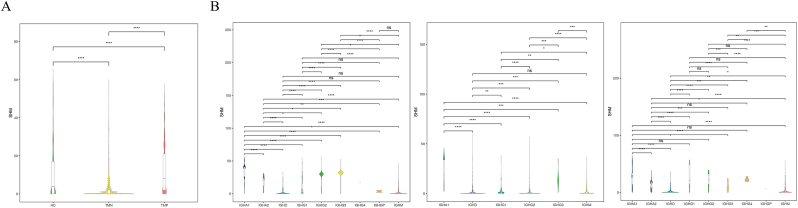
Various immunological indicators of patients' immune function. A: Overview of SHM for the TMP, TMN, and HC groups. B: Classification overview of different subtypes of SHM for the TMP, TMN, and HC groups. The study classified somatic hypermutation (SHM) associated with different antibody subtypes. In the TMP group, the primary significant differences were concentrated within the IgG subtypes, where the IGH2 and IGH3 subtypes exhibited marked increases in SHM compared with the other subtypes.

The SHM analysis revealed significant differences among the groups. The HC and TMN groups presented relatively low mutation rates, whereas the TMP group presented a relatively high mutation rate, particularly in the IGHG group (Fig. 3A and B).

3 Antibody subtype testing revealed that IgG3 dominates the antibody pool in patients with AIGA syndrome

**Fig. 4:**
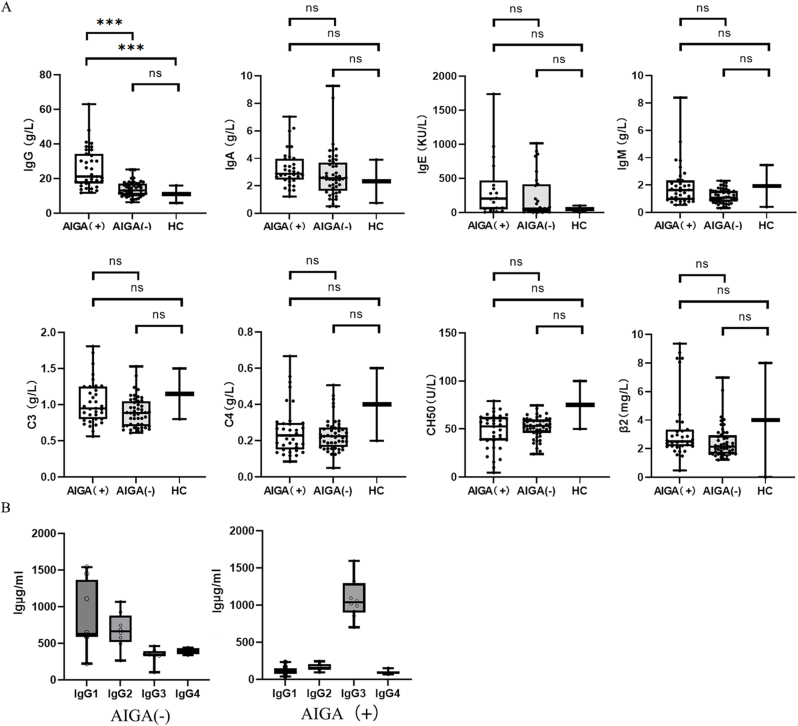
Immunity-related indicator tests. A: Comparison of the compositions or levels of IgG (n = 36, P < 0.05), IgA (n = 35, P > 0.05), IgM (n = 35, P > 0.05), IgE (n = 17, P > 0.05), C3 (n = 35, P > 0.05), C4 (n = 35, P > 0.05), CH50 (n = 35, P > 0.05) and β2 (n = 35, P > 0.05) in different groups of patients in the prospective cohort. B: Distribution of IgG subtypes in AIGAs (n = 10, P < 0.05).

The AIGA(+) group had significantly higher levels of IgG than the other AIGA(−) groups did (p < 0.001), and these levels were also significantly higher than the normal detection limit in adults (<16 g/L). (Fig. 4A). Furthermore, the plasma samples of 10 AIGA(+) and AIGA(−) patients were selected for further analysis of IgG subtypes via ELISA, and the results revealed that the IgG1 and IgG2 subtypes were predominant in AIGA(−) patients. In contrast, the IgG3 subtype appeared to be predominant in AIGA(+) patients (Fig. 4A).

Plasma from retrospectively selected patients was subjected to IFN-γ affinity chromatography to isolate AIGA-enriched fractions. A subsequent IgG subtype-specific ELISA revealed that samples from the AIGA(−) group aligned with those from healthy controls in terms of the IgG distribution, whereas samples from the AIGA(+) group exhibited a predominant IgG3 accumulation (Fig. 4B).

4 AIGAs were successfully isolated and purified from the plasma of patients with AIGA syndrome

**Fig. 5:**
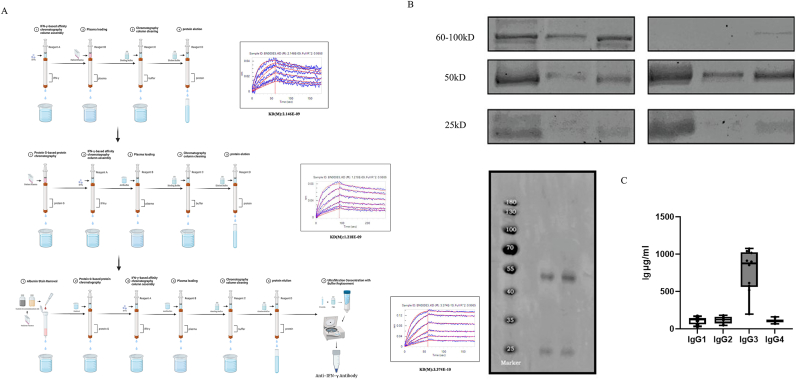
Purification scheme and verification of AIGA components via affinity chromatography. A: Purification scheme and binding strength of AIGAs based on affinity chromatography. This study began with an initial purification scheme utilizing the BLI method as a criterion for assessing purity and potential affinity standards. Through the incorporation of additional processes such as albumin removal technology, IgG enrichment procedures, and multiple rounds of multistep purification, a novel biological marker was successfully isolated and purified while maintaining protein activity as the primary purification objective. B: Validation of the purification results via SDS‒PAGE. C: Mass spectrometry peaks for 50 kDa and 25 kDa bands on an SDS‒PAGE gel after affinity chromatography. D: Proportions of IgG subtypes detected after affinity chromatography (n = 10, P < 0.05).

Our extraction scheme is based on IFN-γ enrichment via an affinity chromatography column. The binding affinity reached a KD(M) value of 3.374 × 10^−10^, and no impurity bands were observed in the 60–100 kDa range on the SDS‒PAGE gel. The binding curve exhibited relative smoothness. The high-performance liquid chromatography‒mass spectrometry analysis of the SDS‒PAGE gel bands enriched at 50 kDa and 25 kDa in the final product revealed that the major component was IgG, with a purity exceeding 95 % (Fig. 5A and B). The intact AIGA extracts were subjected to ELISA for an IgG subtype analysis to validate the results of the plasma AIGA compositional analysis. The results revealed that IgG3 was the predominant isoform present in the intact AIGA product, consistent with the above results for AIGAs in plasma (Fig. 5C).

5 Stronger neutralization activity is the root cause of AIGA functionality

**Fig. 6:**
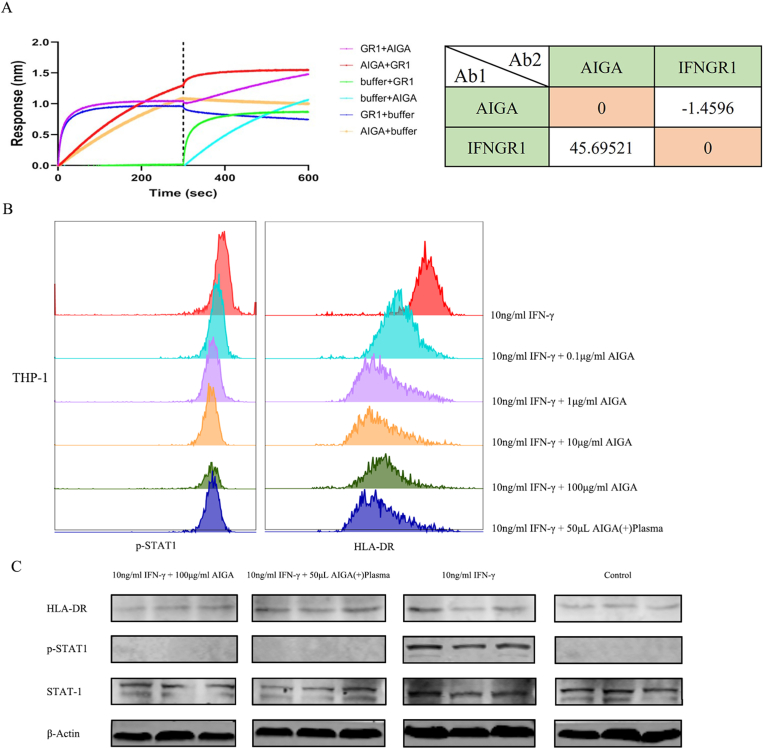
Functional analysis of AIGAs. A: Binding and competitive binding experiments and quantitative analysis of competitive binding. Different types of forward and reverse competitive binding assays were conducted to verify whether the receptor and antibody competitively bind to the same epitope on the surface of IFN-γ. The study revealed that the antibody could effectively compete with IFNGR1 for binding to IFN-γ in vitro, achieving a competition ratio of 45 %, which reached a strong partial competition level. B: Flow cytometry analysis of changes in p-STAT1 and HLA-DR levels in THP-1 cells cultured in the presence of AIGAs. C: Protein imprinting analysis of changes in p-STAT1 and HLA-DR levels in THP-1 cells cultured in the presence of AIGAs.

The results revealed that the competitive effect of AIGAs on IFNGR1 was 45.70 %, whereas the competitive effect of IFNGR1 on AIGAs was −1.460 %. Subsequent experiments in THP-1 cells revealed dose-dependent reductions in p-STAT1 phosphorylation and HLA-DR fluorescence intensity upon AIGA treatment, which was consistent with the observations in patients’ plasma samples. Western blot analysis further confirmed the significant suppression of IFN-γ-induced p-STAT1 and HLA-DR expression by intermediate AIGA concentrations (Fig. 6B and C).

6 A chromatography analysis of AIGAs presents good diagnostic performance in a prospective cohort study

**Fig. 7:**
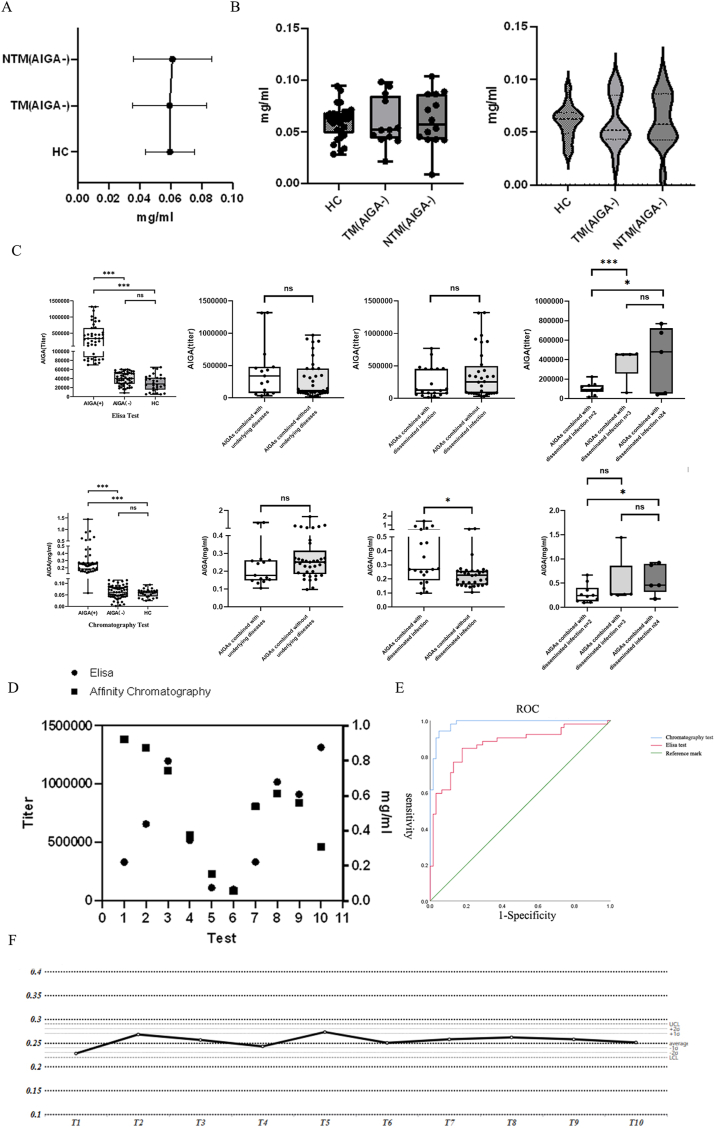
A, B: Analysis of the AIGA contents in the HC, TM, and NTM groups (n = 116, P > 0.05). C: Chart comparing the assays conducted using different methods, ELISA vs. chromatography (n = 116, P < 0.05). The test results of both diagnostic approaches were not significantly influenced by the presence of pre-existing medical conditions. Notably, in the chromatographic assay, patients exhibiting extrapulmonary dissemination presented markedly elevated AIGA concentrations compared with those without dissemination. Furthermore, the diagnostic accuracy of both methodologies progressively increased with an increasing number of disseminated infection sites. D: Comparison of ELISA titers and AIGA concentrations in the same patient. E: ROC curves of the chromatographic and ELISA methods. F: Stability test of clinical detection by affinity chromatography.

This prospective cohort study included AIGA(+) (n = 52), AIGA(−) (n = 62), and healthy control groups (n = 30), and the basic information of the patients was retained for analysis along with plasma and other fluids (Fig. 1B). The basic information of the patients is presented in Supplementary Table 1. A cohort study was conducted to investigate reference values for healthy controls via the AIGA chromatographic extraction method. The results revealed no significant differences in the extracted product levels among patients in the HC, TM, and NTM groups (Fig. 7A and B).

The two assays were validated for an analysis of the population, and both methods were able to differentiate the diseased population. In the stratified analysis, the presence or absence of underlying diseases did not significantly affect the test results of the two diagnostic methods. However, with the chromatographic method, the AIGA concentration was significantly higher in patients with extrapulmonary dissemination than in those without dissemination. Additionally, both methods exhibited improved discriminatory power as the number of dissemination sites increased (Fig. 7C). Ten patients were randomly selected for ELISA and affinity chromatography testing via the AIGA method to achieve more accurate validation. After the measurement standards were pooled, both methods exhibited some deviation in specific patients, with four patients exhibiting significant discrepancies. However, all the results fell within the range of positive detection, and good concordance between the two methods was observed for six patients (Fig. 7 D).

The area under the receiver operating characteristic (ROC) curve for the chromatography method was 0.984 (0.968–1.000), and the area under the curve for the ELISA method was 0.869 (0.799–0.939). The maximum Youden value of the chromatography method was 0.894, corresponding to a sensitivity of 0.942, a specificity of 0.952, and a detection value of 0.124 mg/ml. According to the cutoff value selection principle, the cutoff value was designated at the maximum Youden value. The cutoff value of the chromatography method was 0.1235 mg/ml, and the cutoff value of the ELISA method was a 6.023 × 10^4^ titer (Fig. 7 E). For the diagnosis of AIGAs, DeLong's test and chromatographic tests, which were performed through ROC curves and AUCs, were significantly superior to the ELISA test (p = 0.0018). A comparison of the chromatographic and ELISA methods for AIGA detection via McNemar's equation revealed significant differences in detection sensitivity (P < 0.05) and in detection specificity (P < 0.05) when the expected sample size was achieved (Supplementary Tables 1 and 2). A control chart was constructed by conducting 10 standard tests on individual patient plasma samples to determine the consistency of the method. The errors for T1–T10 fluctuated within two standard deviations (Fig. 7 F).

## 8 Discussion

In this study, we constructed and analyzed the V(D)J immune repertoire of patients with AIGA syndrome. An abnormal antibody library characterized by the IgG3 subtype among IgG isoforms was found. A tandem affinity chromatography-based method was proposed for the isolation and purification of AIGAs. The purified AIGAs reached an affinity of 33 nM for IFN-γ and were confirmed to compete better with the IFN-γ receptor, and functional validation confirmed this interaction. The assay parameters were validated using blood-based AIGAs as a biomarker in a prospective cohort, which showed 0.942 sensitivity and 0.952 specificity. Therefore, chromatography is clearly superior to ELISA in the detection of AIGAs.

In the study by Lin et al., IgG was found to be the major component of antibodies, which was confirmed through the successful isolation of monoclonal AIGAs from NTM patients [5]. Furthermore, a B-cell gene characteristic analysis revealed that IgG is a major component of AIGAs. This finding has been corroborated by prospective cohort studies, providing a theoretical basis for the extraction of self-targeting antibodies. IgG consists of four isoforms, with the IgG4 and IgG1 isoforms being the most common, and IgG3 has also been reported in some patients. Our study revealed that anti-IFN-γ antibodies are predominantly associated with the IgG3 subtype. This difference may be due to differences in the diseases of the study carriers; reports indicate that the IgG4 and IgG1 isoforms in AIGAs have undergone antibody class switching. Further studies are needed to investigate subtype changes caused by differences in the specific source of infection [3,21,22].

The detection methods for AIGA syndrome are limited and lack the ability for early detection, which results in longer treatment times and a higher risk of recurrence for patients with infections caused by TM or NTM pathogens [23,24]. The diagnostic methods rely on detecting the neutralizing capacity for IFN-γ in patient plasma and the level of IFN-γ [25,26]. However, significant controversy exists regarding the use of IFN-γ as a diagnostic marker. Qiu et al. [27], Chen et al. [28], as well as our own research reported no difference in IFN-γ levels between patients with TM or NTM infections and those with AIGA syndrome [23,29]. Attempts to stimulate IFN-γ release have revealed that patients with high-titer AIGAs presenting with a disseminated NTM infection have negative reactions. This outcome is because the AIGAs completely mask the detection of IFN-γ, either because of the strong competitive binding of the selected B27 antibody or differences in binding sites. Various methods have been used for screening, including ELISA, chip-based techniques, protein arrays, and fluid phase analysis [30,31]. Clinical screening methods, such as ELISA, include the direct application of serum or plasma for detection and have good negative predictive values and positive predictive values [32]. However, these methods are limited in their ability to identify negative and positive results, are restricted by the binding of antibodies and substrates, are unable to reflect the diversity of the AIGA composition, and can detect only specific structural AIGAs.

In the 1990s, A Turano [33] and A Caruso [34] performed affinity purification of plasma from HIV-positive patients. However, owing to technical limitations, they were unable to enrich and further purify the products, making a determination of their purity and composition difficult. In contrast, based on the guidance from BLI, we obtained high-purity AIGAs from the plasma of *Malassezia* folliculitis patients who were HIV-negative. The overall affinity of the AIGAs reached 10^−10^, which is almost identical to the binding level of the monoclonal antibodies currently extracted, indicating that this multiclonal antibody reflects the population of AIGAs [5]. Based on these studies and the novel AIGA biomarkers we discovered, we propose a simple affinity chromatography-based approach that provides excellent sensitivity and specificity. The corresponding sensitivity of the chromatographic method was 0.942, the corresponding specificity was 0.952, and the cutoff value was 0.124 mg/ml. The sensitivity and specificity of the chromatographic method differed significantly from those of the ELISA method (DeLong test, P = 0.0018). The core principle is to obtain high-purity AIGAs through consecutive purification steps, with the added benefits of simplicity and scalability. From a cost perspective, stability testing indicated that a single chromatography column remains functional for up to 10 cycles, reducing the per-sample cost to below $15. Additionally, all buffers used in the study are common and fully customizable, avoiding reliance on proprietary formulations. Compared with ELISAs, which suffer from increased costs due to unclear commercial antibody compositions and market-driven procurement challenges, chromatographic methods reduce the per capita costs by nearly one-third. Owing to the high prevalence of AIGA syndrome in Southeast Asia, a combination of portable chromatography columns and syringes can easily be used to complete the entire detection process. The time can be controlled within 3 h. Compared with the ELISA method, the detection time is similar. The detection throughput can be increased by stacking chromatography columns for high-throughput screening. In economically developed regions, the use of protein purification systems such as an AKTA instrument enables one-step consecutive purification and UV-based protein concentration detection, further improving stability.

Our study also has several limitations. In the clinical testing protocol, false-positive and false-negative results were observed, which may be attributed to the inherent errors associated with manual experimental procedures. We plan to enhance the prospective screening of patient cohorts and develop automated testing protocols utilizing the AKTA system to address this limitation. Additionally, the small sample size and lack of longitudinal AIGA monitoring data remain to be resolved. Regional bias further impacted the accuracy of our findings. Future research should focus on conducting larger-scale, comprehensive international multicenter studies to mitigate these limitations.

In summary, we described the changes in humoral immunity in patients at the genetic level, confirmed the clonal distribution of AIGAs through various methods, and isolated and purified highly active and pure polyclonal AIGAs. Importantly, we have provided an absolute quantitative method for detecting AIGAs. Through prospective clinical cohort studies, we demonstrated the high sensitivity and specificity of this method. Its simple procedure and low cost make it highly promising for application in Southeast Asia, where AIGA syndrome is prevalent.

## 9 Conclusions

This study identified AIGA as a humoral immunity biomarker and developed a quantitative detection method with high sensitivity/specificity. The assay was validated in clinical cohorts, and its the simplicity and low-cost design will increase accessibility in Southeast Asia, facilitating the early diagnosis and management of AIGAs.

## CRediT authorship contribution statement

**Xidong Wang:** Formal analysis, Data curation, Conceptualization. **Feng Ye:** Funding acquisition, Formal analysis. **Hongling Liu:** Writing – original draft, Methodology, Data curation. **Shaoqiang Li:** Supervision, Software. **Jinglu Yang:** Resources, Data curation. **Xue Yu:** Resources, Project administration. **Yilei Hui:** Formal analysis. **Yongming Li:** Methodology, Investigation. **Yangqing Zhan:** Investigation, Data curation. **Yan Wang:** Data curation. **Jing Liu:** Resources. **Zhengtu Li:** Writing – review & editing.

## Ethics statement

Ethical approval for the study protocol was granted by the Ethics Committee of the First Affiliated Hospital of 10.13039/100009659Guangzhou Medical University (number 2019–20; Guangzhou, China). Written informed consent was obtained from each participant. Our studies were performed in accordance with relevant guidelines and regulations.

## Consent for publication

Written informed consent was obtained from the patients and their families for the publication of this case report and any accompanying images.

## Availability of data and materials

At the request of the Ethics Committee, the datasets generated and/or analyzed during the current study are available from the Specialist Big Data Management Platform of China, https://copd.tp-data.com/v3/index.html. The accompanying key can be obtained by contacting tu276025@gird.cn.

## Authors’ information

Xidong Wang, Feng Ye, Hongling Liu and Shaoqiang Li are considered co-first authors.

## Funding

National Natural Science Foundation of China (82470008,82270007,82370011),Noncommunicable Chronic Diseases-National Science and Technology Major Project (2023ZD0506200).

## Declaration of competing interest

No potential conflict of interest was reported by the authors.

## Data Availability

Data will be made available on request.
