# Supplementary material for: Characteristics of polyclonal anti-interferon-gamma autoantibodies and novel diagnostic strategies: A prospective cohort study of new biomarkers
Source: J Transl Autoimmun. 2025 May 15;10:100292. doi: 10.1016/j.jtauto.2025.100292 (PMC12143649; doi:10.1016/j.jtauto.2025.100292)
Supplement: Multimedia component 1 [file mmc1.docx]

**Supplementary Methods**

**Patient enrollment and specimen collection**

The research subjects enrolled in the prospective cohort of AIGA syndrome patients were from a multicenter, prospective clinical study in China (ChiCTR2000029306). The patients included were those who were diagnosed with *Talaromyces marneffei* infection and were divided based on the presence of AIGA syndrome. The time range was from January 1, 2020, to December 31, 2023. All patients provided informed consent. Clinical samples, including serum and blood treated with EDTA anticoagulant, were obtained in accordance with routine clinical procedures. The samples were stored between 2°C and 8°C for no more than 48 hours until they were ready for shipment to the central laboratory for processing.

**Inclusion and exclusion criteria**

***Talaromyces marneffei infection***

The inclusion criteria were as follows: 1) age ≥ 18 years; 2) negative HIV test; 3) clinical or (and) imaging manifestations of *Talaromyces marneffei* (*T. marneffei*) infection; and 4) clinically confirmed *T. marneffei* infection via a microbiological or pathological examination. Furthermore, clinically confirmed patients needed to meet any of the following criteria: 1. positive microscopic examination or culture of specimens from sterile sites; 2. direct microscopy and positive fungal culture of skin smear or biopsy, blood, sputum and lymph node, pus, bone marrow, pleural fluid, ascites and cerebrospinal fluid samples; and 3. positive mNGS tests for sputum, percutaneous lung puncture tissue, and bronchoalveolar lavage fluid (BALF) from bronchoscopic biopsies of lung tissue. On the other hand, the exclusion criteria included 1) age < 18 years; 2) positive HIV test; and 3) unwillingness to participate or unwillingness to provide a signed informed consent form.

***Nontuberculous mycobacteria infection***

The patients presented with respiratory and/or systemic symptoms and demonstrated characteristic chest imaging findings including cavitary shadows, multifocal bronchiectasis, and multiple small nodular lesions. After exclusion of other pulmonary diseases and verification of sample integrity (ensuring absence of exogenous contamination), the diagnosis was established when at least one of the following criteria was met. For clinical confirmation, patients were additionally required to fulfill any of the following conditions: (1) 2 sputum samples sent separately for testing with positive NTM culture and identification of the same causative organism and/or NTM molecular biological testing for the same causative organism; (2) 1 positive NTM culture and/or molecular biological testing of bronchial lavage or bronchoalveolar lavage; (3) characteristic histopathological changes indicating mycobacteriosis found on lung biopsy via bronchoscopy or other routes (granulomatous inflammation or positive antacid staining) and 1 positive NTM culture and/or molecular biological test; and (4) characteristic histopathological changes indicating mycobacteriosis (granulomatous inflammation or positive antacid staining) detected by bronchoscopy or other routes of lung biopsy and 1 or more positive NTM cultures and/or molecular biological tests in sputum specimens, bronchial lavage fluid, or bronchoalveolar lavage fluid. On the other hand, the exclusion criteria included 1) age < 18 years; 2) a positive HIV test; and 3) unwillingness to participate or unwillingness to provide a signed informed consent form.

***Anti-IFN-γ autoantibody (AIGA) syndrome***

AIGA syndrome patients were screened from the above patients according to the following criteria:

1. Anti-IFN-γ autoantibody neutralization experiment titer>1:100;

2. Anti-IFN-γ autoantibody ELISA test titer >60000;

3. The antibody was detected to have neutralizing activity (flow cytometry for Stat-1).

Control groups included patients with both of these diseases who had negative AIGA-related tests and were in a stable condition (≥3 months of treatment).

**The single-call test involving most of the 10×Genomic immune repertoire and data analysis**

***PBMC isolation***

The PBMCs were isolated by using a density gradient centrifugation. Briefly, a blood sample from an ethylenediaminetetraacetic acid-anticoagulated tube was added to Ficoll Paque plus density solution and then centrifuged according to the manufacturer’s protocol.

***Single-cell 5’ RNA sequencing***

After the PBMCs were pooled, the suspension was loaded onto the Chromium platform (10x Genomics). Single-cell 5’ RNA sequencing was performed as described in a previous publication. Briefly, we profiled thousands of genes at the single-cell level by barcoding mRNAs at the 5’ end for the unbiased characterization of cell types and cell states.

***BCR profiling***

The samples were processed using the Chromium single-cell V(D)J reagent kit according to the protocol provided by 10x Genomics. We simultaneously profiled the immune repertoire (BCR) and gene expression from the same cell to enable the correlation of the clonotype with the corresponding cellular subtype.

***Data analysis***

All raw data were processed through the CellRanger pipeline (10x Genomics), which allows demultiplexing, alignment, filtering, barcode counting, UMI counting, and generation of cell × barcode matrices. Unsupervised clustering and visualization were performed with R and t-distributed stochastic neighbor embedding (tSNE), respectively. Marker genes were defined as those with a fold change and adjusted p <0.05. The cell clusters were annotated using canonical cell markers. For the BCR clonotype analysis, we used the CellRanger pipeline. According to the definition of 10x Genomics, “clonotypes are defined as a set of cells with the same CDR3 sequence in their V(D)J variable regions”.

**Isolation and purification of AIGAs with chromatography**

***Assembly of AIGA affinity chromatography columns***

The HiTrap NHS-activated mixture (Cytiva, 1 ml) was assembled and connected to a peristaltic pump, and the solution was acidified with 6 ml of 4°C acidification solution (ECOTOP | ED-8774-500 ml) (intracolumn pressure less than 5 bar [0.5 MPa] (70 psi), all operations should be performed with an intracolumn pressure less than 5 bar [0.5 MPa] (70 psi), while 1 mg of human IFN-γ (Sinobiological, 11725-HNAE) was dissolved in 2 ml of coupling buffer (ECOTOP | ED-8703-500 ml). Immediately after acidification, 1 ml of a 0.5 mg/ml IFN-γ solution was injected and incubated for 20 min. The inactivation and removal of unbound IFN-γ were achieved by 3 repeated washes with 5 ml of blocking buffer (ECOTOP ED-8704-500 ml) and wash buffer (ECOTOP ED-8705-500 ml).

***Isolation and purification of polyclonal autoantibodies***

EDTA anticoagulated tubes containing patients’ blood samples were centrifuged at 800 × g for 5 min to extract the upper plasma layer, and the supernatant was removed by the centrifugation of the plasma at 12000 × g for 5 min. Albumin was removed using the Albumin Serum Removal Kit (Thermo Scientific™, 85160) with binding buffer (ECOTOP ED-8706-500 ml), elution buffer (ECOTOP ED-8707-500 ml) and HiTrap Protein G HP (Cytiva, 1 ml) for the enrichment of IgG components and pH correction, and finally, the polyclonal AIGA solution was obtained by separation and purification using an assembled AIGA affinity chromatography column. Biolayer interferometry was used for affinity and competitive binding experiments.

**Identification of the AIGAs**

A gradient dilution of the bovine serum albumin (BSA) standard was used to prepare the BCA working solution. The protein standard and the protein sample to be tested were added to the microtiter plate or test tube at each dilution, and the BCA working solution (the proportion of was determined according to the instruction manual of the kit) was added and mixed. After the samples were sealed at 37°C for 30–60 min, they were cooled to room temperature, and the absorbance value at 562 nm was measured using a blank as a control. The absorbance at 562 nm of each standard and protein sample was measured by subtracting the average absorbance at 562 nm of the blank standard from the absorbance at 562 nm of the sample or standard.

**Functional verification of the AIGAs**

***The binding force of the AIGAs***

Competitive Binding Assays Based on Biological Layer Interferometry (BLI)

The competitive binding of antibodies was performed on a ForteBio Octet Red96 system (Pall Forte BioCorporation, Menlo Park, CA) using a tandem-format sorting assay. Biotinylated proteins were loaded into the sensor (ForteBio, Cat. 18-5019). The sensor was then exposed to 30 µg/mL primary antibody or PBST for 300 seconds and then to 30 µg/mL secondary antibody for 300 seconds.

Biolayer interferometry (BLI)-based affinity test

The chip was mounted according to the standard operating procedures of the OpenSPRTM instrument, activated, the functionalization of the chip surface was completed, and the ligand was prepared. Ligands for immobilization were diluted with activation buffer and injected, and the baseline was observed for 5 min to ensure stability. A high concentration of analyte was injected to confirm ligand activity and to confirm the approximate maximum binding capacity of the surface. The flow rate was increased to 150 µL/min, and the appropriate regeneration buffer was injected to remove the analyte.

***Flow Cytometry***

First, THP-1 cells were cultured, and 10 ng/ml IFN-γ was selected as the stimulus. IFN-γ was mixed with different concentrations of AIGAs and incubated for 10 min for binding. A total of 1*10^5^ THP-1 cells were added to each well of a 24-well plate, and the IFN-γ and AIGA mixtures were added to the cells at different ratios. The cells were incubated at 37°C in a 5% CO_2_ incubator for 4 h, and brefeldin A (MCE, HY-16592) was added according to the detection index. THP-1 cells were washed and stained with an anti-HLA-DR FITC antibody (340688, BD) for 15 min. Fixable viability stain 510 (340688, BD) was used for staining, and the mixture was incubated for 15 min. The cells were incubated with the reagents from the fixation/permeabilization kit (55470, BD)for 15 min. A permeabilization kit (554714, BD) was used to permeabilize the cells on ice for 30 min. Intracellular staining was performed with a PE anti-phospho-STAT1 (Tyr701) antibody (052919, Biolegend) and at 4°C for 30 min. The cells were subsequently washed and then analyzed using an LSR Fortessa X-20 flow cytometer and Treestar FlowJo v10.6.2 software.

***Western blot***

The total protein concentration was measured, and the proteins were boiled, denatured and allowed to reach room temperature. The electrophoresis time was 2 hours, and the voltage was 120 V. After completion, the membrane was transferred to 60 V. After transfer, the membrane was stained with 1× Ribonin staining solution for 5 minutes. The membranes were stained with 1× Rejuvenation Red for 5 minutes and decolorized. The primary antibody was diluted 1000-fold with TBST (in a 15 ml centrifuge tube), the protein membrane was placed face down on the antibody surface, and the mixture was incubated for 1 hour at room temperature. The membrane was washed twice with TBST on a decolorizing shaker at room temperature for 10 minutes each, washed once with TBS, and allowed to stand for 10 minutes. The secondary antibodies were diluted in the same way, incubated with the membrane for 1 hour at room temperature. The membrane was washed twice with TBST on a decolorizing shaker at room temperature for 10 minutes each, washed again with TBS and incubated for 10 minutes for the reaction to occur.

***ELISAs for IgG and IFN-gamma***

The Human IgG1 Elisa Kit, Human IgG2 Elisa Kit, Human IgG3 Elisa Kit, and Human IgG4 Elisa Kit (BMS2092 BMS2093 BMS2094 BMS2095, Invitrogen) were used to test the AIGA crude extract (IgG enrichment was not performed). IFN-γ was added as a substrate to a transparent 96-well plate and blocked, and a B27 antibody (AEAO49Hu, Cloud-clone) was used as the detection antibody for AIGA, which was detected by absorbing light at 450 nm on a Varioskan LUX automated zymography instrument (Thermo Fisher).

***Statistical analysis***

The comparison of differences in sensitivity between two relevant dichotomous samples was used as a design principle, and the two-sided McNemar test was used as a statistical method.

The following calculation formula was used:

**
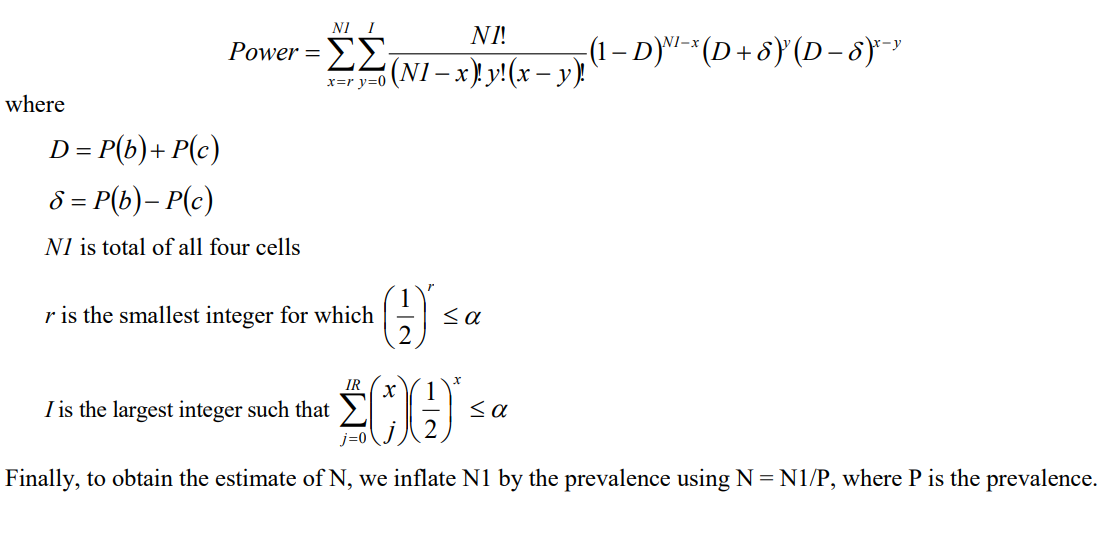
**

Solve for: Sample Size, Alternative Hypothesis: Two-Sided, Power (Grasp): 0.8, Alpha (Level of Significance): 0.05, P (Prevalence) (Expected prevalence of AIGA in Malniffy Basket Mycobacterial Disease or Non-Tuberculous Mycobacterial Disease). 0.5, Se1 (Sensitivity of Test 1) (Expected Sensitivity of Chromatography): 0.95, Se2 (Sensitivity of Test 2) (Expected Sensitivity of Elisa): 0.78, D (Portion Discordant) (Inconsistent Pairwise Ratio of the two tests): 0.19.

The results revealed that a sample size of N=102 subjects could be 80% sufficient to detect a difference of 0.17 between the two diagnostic tests, with sensitivities of 0.95 and 0.78, respectively. This procedure was performed using a two-sided McNemar test with a significance level of 0.05. The prevalence of disease in the population was 0.5. The proportion of inconsistent pairs was 0.19.
