# Supplementary material for: Characteristics of polyclonal anti-interferon-gamma autoantibodies and novel diagnostic strategies: A prospective cohort study of new biomarkers
Source: J Transl Autoimmun. 2025 May 15;10:100292. doi: 10.1016/j.jtauto.2025.100292 (PMC12143649; doi:10.1016/j.jtauto.2025.100292)
Supplement: Multimedia component 2 [file mmc2.docx]

Supplementary Table 1: Basic information of the patients in the prospective clinical cohort

| Clinical indicator | AIGA(+)  (N=52) | TAIGA(-)  (N=30) | NAIGA(-)  (N=32) | HC  (N=30) | P value |
| --- | --- | --- | --- | --- | --- |
| Age (mean±SD) | 54.3±12.8 | 52.5±15.4 | 54.3±13.5 | 49 | P>0.05 |
| Sex (number (% male)) | 31 (59.6%) | 19 (66.6%) | 12 (37.5%) | 15 (50.0%) | P>0.05 |
| Comorbid diseases (% comorbid (total number)) | 36.5% (N=41) | 50% (N=30) | 87.50% (N=32) | / | / |
| Extrapulmonary organ involvement (combined % (total number)) | 48.7% (N=41) | 3% (N=30) | 0% (N=32) | / | / |

Note: "/" indicates not detected.

Supplementary Table 2: Validation of the sensitivity and specificity of the chromatographic assays

| Confusion matrix (math.) | | Real classification | |
| --- | --- | --- | --- |
|  |  | Negative | Positive |
| Detection classification | Negative | 59 | 3 |
|  | Positive | 3 | 49 |

TPR = 0.942

TNR = 0.952

Supplementary Table 3: Chi-square test of differences in sensitivity and specificity

The McNemar test was used to compare the performance of the two classification models based on the paired sample classification outcomes (e.g., correct/incorrect) and is suitable for comparing binary outcomes. Here, we dichotomized the data obtained from the two detection methods into negative and positive categories, enabling a qualitative assessment of sensitivity and specificity in this scenario.

A: A chi-square test for differences in sensitivity

| McNemar's test | | Chromatography | |
| --- | --- | --- | --- |
|  |  | Negative | Positive |
| ELISA | Negative | 2 | 9 |
|  | Positive | 1 | 40 |
|  | Number of active cases |  | 52 |
|  | Precise significance (bilateral) | | 0.021^a^ |

Note: For all patients in the AIGA(+) group who participated in the test, McNemar's test was dichotomized by the results of the chromatography test versus the ELISA. a. Binomial distributions were used.

| McNemar's test | | Chromatography | |
| --- | --- | --- | --- |
|  |  | Negative | Positive |
| ELISA | Negative | 48 | 1 |
|  | Positive | 11 | 2 |
|  | Number of active cases |  | 62 |
|  | Precise significance (bilateral) | | 0.006^a^ |

Note: For all patients in the AIGA(-) group who participated in the test, McNemar's test was dichotomized by the results of the chromatography test versus the ELISA. a. Binomial distributions were used.
