# Supplementary material for: Characteristics of polyclonal anti-interferon-gamma autoantibodies and novel diagnostic strategies: A prospective cohort study of new biomarkers
Source: J Transl Autoimmun. 2025 May 15;10:100292. doi: 10.1016/j.jtauto.2025.100292 (PMC12143649; doi:10.1016/j.jtauto.2025.100292)
Supplement: Multimedia component 3 [file mmc3.docx]

| Abbreviation | Full Term |
| --- | --- |
| AIGA | Anti-interferon-γ autoantibody |
| AIGAs | Anti-interferon-γ autoantibody syndrome |
| IFN-γ | Interferon-gamma |
| IFNGR1 | Interferon gamma receptor 1 |
| BLI | Biolayer interferometry |
| ELISA | Enzyme-linked immunosorbent assay |
| TM | *Talaromyces marneffei* |
| NTM | Nontuberculous mycobacteria |
| IgG | Immunoglobulin G |
| CDR | Complementarity-determining region |
| KD(M) | Dissociation constant of 10 nM |
| EDTA | Ethylenediaminetetraacetic acid |
| ROC | Receiver operating characteristic |
